# Development and validation of an Arabic questionnaire to assess psychosocial determinants of eating behavior among adolescents: a cross-sectional study

**DOI:** 10.1186/s41043-017-0086-0

**Published:** 2017-04-07

**Authors:** Leila Itani, Hanadi Chatila, Hani Dimassi, Fikrat El Sahn

**Affiliations:** 1 Faculty of Health Sciences, Department of Nutrition and Dietetics, Beirut Arab University, Riad El-Solh, Beirut, 1107 2809 Lebanon; 2 Doctoral School of Literature, Humanities and Social Sciences, Lebanese University, Sin El Fil, Beirut, Lebanon; 3 Faculty of Education, Department of Science Education, Lebanese University, Furn El Shebback, Beirut, Lebanon; 4 School of Pharmacy, Lebanese American University, Chouran, Beirut, 1102 2801 Lebanon; 5 High Institute of Public Health, Alexandria University, 65 St., Al-Horeya Road, Alexandria, Egypt

**Keywords:** Questionnaire, Psychosocial determinants, Knowledge, Attitude, Practices, Social norms, Self-efficacy

## Abstract

**Background:**

There is a scarcity of studies that evaluate the psychosocial determinants of eating behavior among adolescents in the Eastern Mediterranean region. The availability of such data is limited by the lack of valid culturally appropriate tools. The current study aims to develop and validate an Arabic questionnaire that measures psychosocial determinants of eating behavior among adolescents.

**Methods:**

A cross-sectional study was carried out to validate a five-scale questionnaire developed to measure nutrition-related knowledge, attitude, practices, and self-efficacy and social norms. Content validity was assessed by Lawshe’s method, factor analysis was used to assess construct validity, and Cronbach’s α was used to test internal consistency. Temporal stability was assessed by test–retest reliability. A random sample of public and private school students participated in the validation study.

**Results:**

All the five scales demonstrated excellent content validity (content validity ratio, CVR ≥0.778). Factor analysis revealed several dimensions for each scale. Cronbach’s *α* for the identified dimensions or subscales ranged between 0.495 and 0.809 indicating acceptable internal consistency. Cronbach’s α for the total scales ranged between 0.759 and 0.836. Test–retest analysis revealed good temporal stability (intraclass correlation, ICC >0.7).

**Conclusions:**

A psychometrically valid tool to measure psychosocial determinants of eating behavior was developed. This tool can serve as a potential instrument for pretest and impact evaluation of ongoing nutrition education interventions and curricula. Based on results obtained from this tool, efficacious modifications can be instilled for nutrition policies and interventions.

**Electronic supplementary material:**

The online version of this article (doi:10.1186/s41043-017-0086-0) contains supplementary material, which is available to authorized users.

## Background

Childhood and adolescence are critical periods of life during which eating behavior and food preferences are established and often persist into adulthood [1–4]. However, children cannot adopt a healthy eating pattern by instinct. They need to be informed and motivated through theory-based nutrition education curricula that target psychosocial determinants of eating behavior, namely knowledge, attitudes, normative beliefs, and self-efficacy [5, 6]. The importance of these determinants in shaping nutrition behavior have been recently demonstrated by a significant body of evidence [7–16]. Furthermore, when these determinants are targeted through successful school-based nutrition education interventions, significant changes in nutrition behavior are achieved [13] alongside positive health-related consequences [16].

To promote nutritional health, educators and stakeholders globally issued the “Nutrition-Friendly Schools Initiative” that call on integration of nutrition education into school curricula [17]. Subsequently, the “Regional Strategy on Nutrition 2010-2019” was issued for the Eastern Mediterranean region to reduce nutrition problems in the region through intervention strategies including school-based nutrition education [18]. Although many countries in the Eastern Mediterranean region have responded to the strategy, there is a scarcity of studies that evaluate the impact of nutrition education in these countries [19] particularly in terms of psychosocial determinants of eating behavior. Moreover, considering the results of dietary studies in the region, apparently education interventions have been ineffective. These studies revealed unhealthy eating patterns particularly among adolescents [19–25]. The identified patterns included skipping breakfast, westernized patterns, decreased fruit and vegetable intake, and increased sweetened beverage intake. Furthermore, these patterns were associated with health risks including overweight and obesity, enlarged waist, deficiencies, and nutrition-related diseases [19, 20, 26–28]. While dietary patterns and their socioeconomic determinants have been described particularly among adolescents [23, 26, 27, 29], data is still lacking on the psychosocial determinants of these patterns specifically in this age group.

Obviously there is shortage of data on psychosocial determinants of eating behavior among adolescents in the region. Furthermore, the role of these psychosocial determinants in defining nutrition behavior or the nutritional status of adolescents in these countries is not well understood, which hinders its use for designing effective education interventions. The availability of such data is limited by the lack of specific culturally appropriate tools that can measure and evaluate these determinants.

This study attempts to develop and determine the reliability and validity of a self-administered questionnaire that shall serve as a valid evaluation tool for assessing psychosocial determinates of eating behavior among Arabic-speaking adolescents. The results from these evaluations will serve as reference data for future evaluations and reformation toward more effective nutrition education curricula and interventions.

## Methods

### Study design

A cross-sectional study was conducted to determine the validity and reliability of the developed questionnaire.

### Questionnaire construction

The questionnaire development and validation was carried out through the following steps:

#### Step 1: construction of the questionnaire items

A literature review of existing questionnaires and dietary guidelines [30–32] was conducted to guide item development. The developed questionnaire consisted of five scales: (1) knowledge, (2) attitude, (3) social norms, (4) self-efficacy, and (5) practices. All food items used in the questionnaire were locally consumed food items. The questionnaire was initially constructed in the English language and then translated to Arabic by the PI to ensure cultural appropriateness. To confirm accurate translation to Arabic, the questionnaire was back translated to English by a translator and the two versions were compared for equivalence. The questionnaire was pilot tested on a group of Lebanese adolescents (*n* = 6), and modifications were introduced accordingly.

#### Step 2: content validity

To establish content validity for the final draft, Lawshe’s method for content validity analysis was used [33]. The questionnaire items were evaluated by a group of nine dietitians as subject matter experts (SME). The validators rated items either as “essential,” “useful,” or “not necessary.” A dichotomy was then created from the three-point rating scale into “essential,” “useful,” and “not necessary” (personal communication in an email to Professor F. Robert Wilson (wilsonfrobert@gmail.com), 25 February 2014). A content validity ratio (CVR) for each item was calculated as described by Lawshe [33]. The revised binomial probability distribution for Lawshe’s critical values were used to exclude items rated as “not necessary” [34]. Consequently, all those items with CVR below the critical value of 0.778 were excluded [34]. A scale content validity index (S-CVI) was calculated for each scale by averaging the CVR for all the retained items in the scale [35, 36]. A S-CVI ≥0.9 would indicate excellent content validity at the level of the scale [35].

After modification, 93 items were retained for all the five scales (Table 1).

**Table 1:** The number of items and sub-items and the maximum and minimum possible score in each scale of the questionnaire

		Item score	
Scale	Number of items	Minimum	Maximum
Knowledge	36	0	127
Attitude	16	16	112
Social norms (normative beliefs)	5	5	35
Self-efficacy (control beliefs)	17	17	51
Practices	19	19	95
Total	93		

#### Step 3: internal consistency and construct validity

To examine the construct validity and internal consistency of the final questionnaire, a random sample of 482 grade 12 students (17–18 years) from nine public schools in Beirut was selected to participate in the study. Only 159 completed the questionnaire. The time needed to complete the questionnaire was 30–40 min.

Construct validity was determined by exploratory factor analysis with varimax rotation. The Kaiser–Meyer–Olkin (KMO) test and the *X* ^2^ Bartlett test of sphericity were used to examine the sampling adequacy and the strength of correlations between each scale item respectively [37]. The number of factors retained was based on the inflection point of the scree plot and the interpretability of factors.

Cronbach’s α and item-to-total correlation was used to measure internal consistency of the scales and how much the items in each scale are interrelated respectively [38, 39]. Cronbach’s α was calculated for total scales, for total subscales, and if an item was removed from a subscale. A good consistency of the scale was defined for Cronbach’s α values between 0.5 and 0.7 based on dimensionality of the scale [40, 41]. However, a lower Cronbach’s α was considered sufficient to indicate consistency for scales with less than 10 items [42]. For item-to-total correlation, a correlation higher than 0.2 suggested that each item has a good correlation with the scale [43]. Items having item-to-total correlation less than 0.2 were retained if Cronbach’s α did not increase upon deleting these items [44].

#### Step 4: test–retest reliability of the final questionnaire

To assess the ability of the questionnaire to measure knowledge, attitude, practices, social norms, and self-efficacy with stability over time, it was administered to a sample of 30 grade 12 students (16 boys and 14 girls) aged 17–18 years, selected randomly from a private and a public school in Beirut. The questionnaire was administered twice with a period of 2 weeks between each sitting. To determine test–retest reliability, a paired *t* test analysis was conducted to compare mean scores at T1 and T2. Pearson correlation coefficient between test scores at T1 and T2 was calculated and a two-way random effect model with consistency intraclass correlation (ICC) was computed [45]. Values of ICC were interpreted as follows: >0.75 was excellent, between 0.40 and 0.75 was fair to good, and <0.40 was poor [46].

#### Scoring of the final questionnaire

Each scale was scored based on the type of items included. For the knowledge section, questions with single response were coded into 0 and 1 for wrong/do not know and correct answers respectively. The diet–disease-association questions consisted of a composite score for each item (yes/no and specify). The yes and no responses for these questions were coded into 0 and 1 respectively. The “specify” items in this section entailed more than one possible answer; thus, the guessing correction factor suggested by Sočan was applied here [47]. Hence, a correct response was scored with 1 point, a missing item with 0, and an incorrect response with −1/(*m* − 1) points, where *m* is the number of alternative responses. So +1 was given for a correct answer and −1, −0.5, −0.33, or −0.25 for wrong answers depending on the number of response options (2, 3, 4, or 5 respectively) [47]. The same guessing correction scoring factors were applied for vitamin/mineral food sources in the knowledge section since these entailed more than one response. Positive attitude and practices items, social norms, and self-efficacy were not recoded, and the responses for each item ranged between 1 and 7. Responses for negative attitude and practices items were recoded inversely on the Likert scale. The scores for each scale or subscale were calculated by summing up the scores for all the scale items and sub-items. The minimum and maximum scores for each scale are shown in Table 1.

### Data analysis

All data were entered, cleaned, and analyzed using SPSS 21 (SPSS Inc., Chicago, IL, USA). Statistical significance was accepted at *p* < 0.05.

## Results

A five-scale questionnaire was developed and validated and assessed for temporal stability.

### Content validity

The content validity ratio (CVR) for each item was either 0.778 or 1, which is in agreement with the critical value set for considering items as content valid [34]. These results indicate that 88.9 to 100% of the validators considered the items as either “essential” or “useful.” The scale content validity index (S-CVI) was 0.988 for the total knowledge scale, 0.958 for the attitude scale, 1 for the social norms scale, 0.977 for the practices scale, and 0.934 for the self-efficacy scale.

### Construct validity

When the responses for the 159 completed questionnaires were analyzed for construct validity by factor analysis, the Kaiser–Meyer–Olkin measures of sampling adequacy (KMO) ranged between 0.805 and 0.907 for the knowledge, attitude, self-efficacy, and practices scales and it was 0.605 for the social norms scale, indicating that the correlations among the items of each scale was sufficiently strong for a factor analysis [37]. Bartlett’s test for sphericity also demonstrated suitability of the data for factor analysis in all the scales (*p* < 0.001). Factor analysis revealed a four-factor solution for the knowledge scale, two factors for the attitude scale, one for the social norms scale, four for the self-efficacy scale, and three for the practices scale. The four subscales for the knowledge scale included (1) macronutrients and diseases association, (2) healthy nutrient sources, (3) energy and nutrient balance, and (4) nutritional deficiencies. For the attitude scale, the two subscales were (1) adherence to dietary guidelines and adequacy and (2) salt, sugar, refined grains, and health. For the self-efficacy scale, the four subscales were (1) lifestyle, (2) healthy snacks, (3) calorie control, and (4) adherence to dietary guidelines. For the practices scales, the three subscales were (1) adherence to dietary guidelines, (2) salt and sugar food choices, and (3) lifestyle and portion size. The factor solutions explained 37.12, 41.05, 57.33, 51.32, and 41.42% of the total variance in each of the knowledge, attitude, social norms, self-efficacy, and practices scales respectively. The factor loadings after varimax rotation for all the scales under study are shown in the Additional file 1. Few items with lower loadings were retained because these items were important for content validity of the scale [37].

### Internal consistency

Item analysis of the five scales under study (knowledge, attitudes, social norms, self-efficacy, and practices) revealed acceptable internal consistency measured by Cronbach’s α and item-to-total correlation. Cronbach’s α for total scale varied between 0.836 and 0.759 for each of the knowledge, attitude, practices, and self-efficacy scales. Cronbach’s α for the four knowledge subscales varied between 0.503 and 0.752; for the two attitude subscales, values were 0.495 and 0.809; for the four self-efficacy subscales, values varied between 0.595 and 0.727; and for the practices subscales, values varied between 0.475 and 0.756. Although few subscales attained low Cronbach’s α, items were retained for the sake of content validity and since these subscales included a small number of items [42]. As for the social norms scale, it was also retained (Cronbach’s *α* = 0.376) since it is a unidimensional scale with small number of items [43, 48–50]. The corrected item-to-total correlation for most items in the different subscales were >0.2, which indicates that each item was correlated with the subscale it belongs to [43]. However, few items with item-to-total correlation <0.2 were retained when Cronbach’s α did change if the item was deleted [44]. Inter-item correlations (data not shown) were also within the acceptable range of <0.8 [44]. Item analysis results of the five scales under study are shown in the Additional file 1.

### Test–retest reliability of the final questionnaire

Results for the test–retest reliability are shown in Table 2. ICC was 0.778 for the knowledge scale, 0.921 for the attitude scale, 0.850 for the social norms scale, 0.848 for the self-efficacy scale, and 0.752 for the practices scale, indicating excellent consistency between the two sittings [46]. Inter-item correlation were statistically significant (*p* < 0.05) between the two sittings. The paired *t* test analysis showed that the mean score did not vary significantly from T1 to T2.

**Table 2:** Mean and standard deviations, Pearson’s correlation coefficients, and intraclass correlation for the scores of each scale at T1 and T2 (*n* = 30)

Scale	Mean scores			Correlation between scores at T1 and T2	Intraclass correlation (ICC)		
	T1	T2	Paired *t* test				
	Mean ± SD		*p* value	Correlation coefficient	ICC	95% CI	Value
Knowledge	68.1 ± 13.9	67.2 ± 15.2	>0.05	0.639*	0.778	0.527–0.896	<0.001
Attitude	82.8 ± 17.9	83.3 ± 21.0	>0.05	0.865*	0.921	0.830–0.964	<0.001
Social norms	24.7 ± 4.5	24.8 ± 6.1	>0.05	0.772*	0.850	0.680–0.930	<0.001
Self-efficacy	41.6 ± 7.9	40.7 ± 9.2	>0.05	0.744*	0.848	0.676–0.929	<0.001
Practices	58.3 ± 11.9	57.0 ± 9.6	>0.05	0.617*	0.752	0.472–0.884	<0.001

* The correlations are significant at *P* < 0.05

## Discussion

A questionnaire was developed to address the need for a valid and reliable tool to measure psychosocial determinants of eating behavior in Lebanon and the region. The questionnaire was constructed based on recently advocated healthy eating guidelines [31]. The food items used in the questionnaire were based on those most commonly consumed by the Lebanese population [51].

The scales for all the constructs measured in the questionnaire met the standard criteria for excellent content validity [35]. This indicates that each scale has an appropriate sample of items for the construct being measured [35]. The content validity ratio and content validity index for the scales studied were in accordance with those obtained for the Caspian-IV study questionnaire items assessing attitude and knowledge of the determinants of under- and overweight among Iranian children [52]. These results were in accordance as well with the finding of Koo et al. [53] in their study on the questionnaire developed to assess KAP toward whole grain among primary school children in Kuala Lumpur, Malaysia.

Construct validity was assessed by factor analysis. The results of factor analysis revealed good correlation between items; however, multidimensionality of the different scales was observed. The observed dimensions or subscales were in parallel with the content of the dietary guidelines examined. The knowledge scale measured awareness about energy and nutrient balance and knowledge of healthy nutrient sources and diet–disease associations. The practices scale measured the adherence to dietary guidelines, limiting unhealthy food choices and control of portion size. The attitude scale measured the judgment of the participants about limiting unhealthy food choices and the adherence to dietary guidelines. The self-efficacy scale measured their confidence in being able to follow a healthy lifestyle, choose healthy snacks, control calories, and follow dietary guidelines about breakfast consumption and fruit and vegetable intake. The social norms scale measured the existence of a role for important others in influencing healthy food choices.

Analysis of item-to-total correlation confirmed that each item belonged to its corresponding subscale. Further analysis of internal consistency using Cronbach’s α revealed an acceptable level of internal consistency for the total scales and subscales identified from factor analysis for the knowledge, attitude, self-efficacy, and practices domains. Although certain subscales had moderate alpha values, Cronbach’s alpha ranging between 0.5 and 0.8 have been reported in the literature [54–57]. Furthermore, values of Cronbach’s alpha less than 0.7 are common for one-dimensional scales with less than 10 items [42, 48] and have been justified. Loewenthal and Cortina justified that the alpha coefficient can be lower if the scale had fewer than 10 items due to the profound effect a small number of items have on the alpha value [42, 49]. Also, the low Cronbach’s α of some subscales could be attributed to the difference in dispersion of the responses to items in the subscales. The tendency of individuals to answer toward the extremes will decrease the spread of responses on each subscale item, thus decreasing the size of sub-item correlations, consequently giving a lower Cronbach’s α [58, 59]. Regarding the social norms scale, the construct has been studied and declared by several health behavior theories as an important predictor of behavior intention and health behavior [60]. Thus, for the sake of content validity and with the presence of the justified theoretical and practical reasoning described above, the items for the social norms scale were retained [42, 49, 60]. Furthermore, the moderate Cronbach’s α for items in each scale or subscale indicate that items were satisfactorily interrelated with little redundancy [61]; thus, each item in each scale would be measuring something different. The low inter-item correlation observed would further indicate lower homogeneity which is preferable particularly for the use with areas of motivation and personality which is the case for this questionnaire [61].

In terms of temporal stability, the scores for all the retained items in the different scales and subscales showed good to excellent stability measured by ICC [46]. The results for the temporal stability of the current scales were in line with the reliability results of the “child nutrition questionnaire” [62], the SCREEN nutrition tool [63], and the ENERGY–child questionnaire [64]. Pearson correlation analysis confirmed the ICC results and were in accordance with those reported for the “school-based nutrition monitoring questionnaire” [65], the “nutrition knowledge questionnaire for obese adults” [66] the “questionnaire to test knowledge and practices of dietitians regarding dietary supplements” [67], the “physical activity questionnaire developed for parents of preschool children in Mexico” [68], and the “questionnaire on dietary fiber-related knowledge” [69].

## Conclusions

The current research study provides the first psychometrically valid and reliable tool for use to assess psychosocial determinants of eating behavior among Lebanese- and Arabic-speaking adolescents in situation analysis. The results from the current study indicate that the scales developed are valid and reliable to measure the corresponding constructs constantly over time. The tool also included items that were satisfactorily interrelated, within each scale or subscale, as measured by Cronbach’s α statistic, with little redundancy. The tool can measure the type and level of an adolescent’s nutritional knowledge as well as their attitude toward healthy eating. Further, it can measure the strength of self-efficacy to abide by healthy eating practices and their current adherence to these practices and the influence of important others on their food choices.

Also, it can serve as a potential instrument for pretest and impact evaluation of ongoing nutrition education interventions and curricula. Based on results obtained, efficacious modifications can be instilled in nutrition policies or ongoing interventions to coin a nutrition-literate citizen, where nutrition literacy encompasses the cognitive and social skills, as well as the ability to gain access to, understand, and use nutrition information and material in ways which promote and maintain good health [70, 71]. Hence, with monitoring and refining nutrition education, children will develop into productive members of society and contribute to national development [72].

## Additional file

Additional file 1: Table S1. Rotated factor loading matrix, Item-to-total score correlation and internal consistency for the Knowledge scale items. (DOCX 33 kb)

## Abbreviations

CVR: Content validity ratio
ICC: Intraclass correlation
KMO test: Keiser–Meyer–Olkin test
S-CVI: Scale content validity index
SME: Subject matter experts
