# Supplementary material for: Development and validation of an Arabic questionnaire to assess psychosocial determinants of eating behavior among adolescents: a cross-sectional study
Source: J Health Popul Nutr. 2017 Apr 7;36:10. doi: 10.1186/s41043-017-0086-0 (PMC5383942; doi:10.1186/s41043-017-0086-0)
Supplement: Additional file 1: Table S1. — Rotated factor loading matrix, Item-to-total score correlation and internal consistency for the Knowledge scale items. (DOCX 33 kb) [file 41043_2017_86_MOESM1_ESM.docx]

**Additional file 1: Table S1.** Rotated factor loading matrix, Item-to-total score correlation and internal consistency for the Knowledge scale items.

|  | Factor 1 | Factor 2 | Factor 3 | Factor 4 | Corrected Item-Total Correlation | Alpha if Item Deleted | Alpha for total scale |
| --- | --- | --- | --- | --- | --- | --- | --- |
| **Knowledge items** |  |  |  |  |  |  |  |
|  |  |  |  |  |  |  | **0.759** |
| ***Macronutrient disease association*** |  |  |  |  |  |  |  |
| 1. Have you ever heard of a health problem that can result from overweight and obesity | **0.750** | 0.202 |  |  | 0.599 | 0.694 |  |
| 1. Have you ever heard of a health problem that can result from eating too much fat? | **0.750** | 0.125 |  |  | 0.570 | 0.701 |  |
| 1. Do you know of any health problem that result from eating too much salt? | **0.625** |  |  | 0.128 | 0.488 | 0.720 |  |
| 1. Do you know of any health problem that result from eating too much cholesterol? | **0.590** | 0.138 |  |  | 0.486 | 0.720 |  |
| 1. Have you ever heard of a health problem that can result from overconsumption of food energy (Calories) and sugar sweetened beverage | **0.545** |  | 0.173 | 0.165 | 0.443 | 0.728 |  |
| 1. Do you know of any health problem that result from eating too much sugar? | **0.496** |  | 0.133 |  | 0.372 | 0.740 |  |
| 1. Do you know of any health problem that result from eating too much saturated fat? | **0.478** | 0.110 | 0.198 |  | 0.418 | 0.732 |  |
| 1. Have you ever heard of a health problem that can result from consuming too little vitamin D | **0.337** |  | -0.254 | 0.325 | 0.219 | 0.767 |  |
| **Total subscale Cronbach’s alpha** |  |  |  |  |  | **0.752** |  |
|  |  |  |  |  |  |  |  |
| ***Healthy nutrients sources*** |  |  |  |  |  |  |  |
| 1. 1 small apple provides how many servings from the fruit group | 0.221 | **0.682** | -0.100 |  | 0.376 | 0.483 |  |
| 1. ¼ loaf of Arabic bread provides how many servings from the bread and cereals group |  | **0.634** |  |  | 0.243 | 0.495 |  |
| 1. One cup (one glass) of milk provides how many servings form the milk group? | -0.253 | **0.589** | 0.151 |  | 0.237 | 0.496 |  |
| 1. 90 g of meat provides how many serving from the meat group | -0.122 | **0.564** | 0.187 | -0.119 | 0.231 | 0.498 |  |
| 1. In the list below the food with high Omega 3 content is |  | **0.430** | 0.166 | 0.202 | 0.307 | 0.481 |  |
| 1. Identify foods in this list that are good sources of the following minerals | 0.191 | **0.415** | 0.126 | 0.321 | 0.361 | 0.412 |  |
| 1. Which of the following food items are low or high in fiber? |  | **0.401** | 0.285 | 0.192 | 0.365 | 0.445 |  |
| 1. In the list below which is the healthier fat to eat: | 0.308 | **0.384** | 0.176 |  | 0.390 | 0.479 |  |
| 1. identify items in this list that are good sources of the following vitamins | 0.207 | **0.379** |  |  | 0.274 | 0.465 |  |
| 1. In the list below the food with the high dietary fiber content is |  | **0.358** | 0.225 | 0.176 | 0.394 | 0.470 |  |
| **Total subscale Cronbach’s alpha** |  |  |  |  |  | **0.503** |  |
|  |  |  |  |  |  |  |  |
| ***Energy and nutrient balance*** |  |  |  |  |  |  |  |
| 1. Which of the following food items are low or high in salt? | 0.191 | 0.208 | **0.711** |  | 0.625 | 0.529 |  |
| 1. Which of the following food items are low or high in fat? | 0.294 |  | **0.695** |  | 0.574 | 0.548 |  |
| 1. Which the following food items are low or high in added sugar? | 0.267 | 0.130 | **0.603** | -0.202 | 0.541 | 0.567 |  |
| 1. Which of the following food items are low or high in Calories |  |  | **0.529** |  | 0.369 | 0.612 |  |
| 1. Which of the following have more saturated fat based on equal portion size |  | 0.194 | **0.405** | 0.114 | 0.293 | 0.636 |  |
| 1. The proportion of energy coming from protein in a balanced daily food ration should be | -0.170 |  | **0.363** | 0.128 | 0.150 | 0.648 |  |
| 1. Which of the following is high in Polyunsaturated fat |  | 0.124 | **0.278** |  | 0.173 | 0.645 |  |
| 1. Have you ever heard of a health problem that can result from starvation (Food energy and protein deficiency? |  |  | **0.272** | 0.206 | 0.168 | 0.644 |  |
| 1. Which of the following is a source of Monounsaturated fat | 0.104 | 0.206 | **0.215** | 0.107 | 0.204 | 0.645 |  |
| 1. Which of the nutrients below have the highest energy content per gram weight | 0.192 |  | **0.192** |  | 0.136 | 0.648 |  |
| 1. The proportion of energy coming from fats (lipid) in a balanced daily food ration should be |  |  | **0.182** | 0.110 | 0.083 | 0.650 |  |
| **Total subscale Cronbach’s alpha** |  |  |  |  |  | **0.631** |  |
|  |  |  |  |  |  |  |  |
| ***Nutritional deficiencies*** |  |  |  |  |  |  |  |
| 1. Have you ever heard of a health problem that can result from consuming too little Zinc? |  |  | 0.175 | **0.764** | 0.516 | 0.548 |  |
| 1. Have you ever heard of a health problem that can result from consuming too little vitamin A (retinol)? |  |  |  | **0.698** | 0.457 | 0.557 |  |
| 1. Have you ever heard of a health problem that can result from consuming too little iodine? |  |  |  | **0.659** | 0.425 | 0.570 |  |
| 1. Have you ever heard of a health problem that can result from consuming too little Iron? | 0.201 | 0.114 |  | **0.471** | 0.335 | 0.603 |  |
| 1. Have you ever heard of a health problem that can result from consuming too little Calcium? | 0.301 | 0.189 |  | **0.466** | 0.379 | 0.583 |  |
| 1. The proportion of energy coming from carbohydrates in a balanced daily food ration should be |  | 0.116 | 0.147 | **0.412** | 0.238 | 0.624 |  |
| 1. Do you know of any health problem that result from eating too much trans-fat? |  | 0.107 | 0.216 | **0.218** | 0.139 | 0.660 |  |
| **Total subscale Cronbach’s alpha** |  |  |  |  |  | **0.631** |  |

Factor loading less than 0.1 were excluded from the table for simplicity

**Table 2**. Rotated factor loading matrix, Item-to-total score correlation and internal consistency for the attitude scale items.

|  | Factor 1 | Factor 2 | Corrected Item-Total Correlation | Alpha if Item Deleted | Alpha for total scale |
| --- | --- | --- | --- | --- | --- |
| **Attitude items** |  |  |  |  |  |
| ***Adherance to dietary guidelines and adequacy*** |  |  |  |  | **0.792** |
| 1. Eating fruits and vegetables regularly on a daily basis will help me prevent disease in the future | **0.694** |  | 0.563 | 0.789 |  |
| 1. Replacing sugar sweetened beverages with water will help me maintain a healthy body weight | **0.686** |  | 0.550 | 0.789 |  |
| 1. Enough dietary fibers can be obtained by eating daily more fruits, vegetables and legumes | **0.675** |  | 0.512 | 0.790 |  |
| 1. Choosing more often foods low in fat will help maintain a healthy body weight | **0.666** |  | 0.550 | 0.787 |  |
| 1. A nutritionally adequate diet is essential to support proper growth and good health | **0.661** |  | 0.518 | 0.790 |  |
| 1. Fruits and vegetables are good substitutes for junk foods | **0.568** |  | 0.491 | 0.791 |  |
| 1. What you eat and drink cannot affect your chances of getting any diseases like heart disease and cancer | **0.552** |  | 0.388 | 0.803 |  |
| 1. Eating daily a variety of foods from all the food groups will provide all vitamins and minerals i need to grow and stay healthy | **0.481** |  | 0.411 | 0.799 |  |
| 1. Eating less fatty meat or animal products and replacing them with legumes and fish will help me maintain good health | **0.474** |  | 0.412 | 0.799 |  |
| 1. Choosing more often foods low in saturated fat and cholesterol will help me prevent disease in the future | **0.412** | 0.306 | 0.407 | 0.800 |  |
| 1. Skipping meals such as breakfast and lunch makes it hard for me to do well in classes | **0.411** | 0.394 | 0.443 | 0.798 |  |
| 1. Balancing calories intake with physical activity will help me maintain a healthy body weight | **0.349** |  | 0.354 | 0.803 |  |
| **Total subscale Cronbach’s alpha** |  |  |  | **0.809** |  |
| ***Salt ,sugar, refined grains and health*** |  |  |  |  |  |
| 1. Using salt and salty foods only in moderation will help me prevent heart disease and hypertension in the future |  | **0.752** | 0.393 | 0.317 |  |
| 1. Eating less from sweetened foods will help me maintain good health |  | **0.615** | 0.295 | 0.419 |  |
| 1. To maintain a healthy body weight, whole grain foods are good substitute for refined grain products |  | **0.576** | 0.318 | 0.406 |  |
| 1. Eating smaller portions from energy dense foods will help me maintain a healthy body weight |  | **0.416** | 0.172 | 0.532 |  |
| **Total subscale Cronbach’s alpha** |  |  |  | **0.495** |  |

Factor loading less than 0.3 were excluded from the table for simplicity

Item 7 was coded inversely before factor analysis

**Table 3**. Rotated factor loading matrix, Item-to-total score correlation and internal consistency for the Social norms items.

|  | Factor 1 | Corrected Item-Total Correlation | Alpha if Item Deleted | Alpha for total scale |
| --- | --- | --- | --- | --- |
| **Social norms items** |  |  |  |  |
|  |  |  |  | **0.376** |
| 1. I am expected to eat smaller portions at fast food restaurants to maintain healthy body weight | **0.724** | 0.254 | 0.262 |  |
| 1. Most people important to me think I should eat less fatty food to maintain good health | **0.678** | 0.273 | 0.247 |  |
| 1. My peers think that fruits and vegetables are important to health | **0.521** | 0.176 | 0.338 |  |
| 1. When I am with my friends it becomes difficult for me to avoid eating junk food | **0.389** | 0.147 | 0.356 |  |
| 1. People important to me think I should eat foods from all the food groups | **0.254** | 0.096 | 0.397 |  |
| **Total subscale Cronbach’s alpha** |  |  | **0.376** |  |

Factor loading less than 0.1 were excluded from the table for simplicity

**Table 4**. Rotated factor loading matrix Item-to-total score correlation and internal consistency for the Self-efficacy items.

|  | Factor 1 | Factor 2 | Factor 3 | Factor 4 | Corrected Item-Total Correlation | Alpha if Item Deleted | Alpha for total scale |
| --- | --- | --- | --- | --- | --- | --- | --- |
| **Self-efficacy items** |  |  |  |  |  |  |  |
| ***Life style*** |  |  |  |  |  |  | **0.836** |
| 1. Drink a glass of my favorite natural fruit juice as a refresher during the day instead of sweetened beverages | **0.692** |  | 0.145 | 0.217 | 0.503 | 0.637 |  |
| 1. Ask my mom to prepare food with a little bit of oil or fat | **0.632** | 0.140 | 0.124 |  | 0.458 | 0.650 |  |
| 1. Ask my mom to buy for me low fat milk and dairy products | **0.607** | 0.229 |  | 0.158 | 0.466 | 0.648 |  |
| 1. Order half the regular portion from ice cream | **0.576** |  | 0.210 |  | 0.378 | 0.675 |  |
| 1. Use a spoon or measuring cup to pour corn flakes instead of pouring from the whole box | **0.574** | 0.216 | 0.328 |  | 0.486 | 0.641 |  |
| 1. Drink water instead of bottled juice and soda most of the time | **0.420** | 0.297 | -0.168 | 0.226 | 0.304 | 0.702 |  |
| **Total subscale Cronbach’s Alpha** |  |  |  |  |  | **0.699** |  |
|  |  |  |  |  |  |  |  |
| ***Healthy snacks*** |  |  |  |  |  |  |  |
| 1. Snack on the vegetable I like instead of chips and chocolate | 0.143 | **0.797** |  | 0.211 | 0.606 | 0.612 |  |
| 1. Snack on the fruit I like instead of candy or salty snacks | 0.360 | **0.713** |  | 0.248 | 0.564 | 0.637 |  |
| 1. Eat half a chocolate bar for a snack instead of eating the whole bar |  | **0.611** | 0.492 | -0.142 | 0.384 | 0.744 |  |
| 1. Eat only a handful of chips instead of the whole bag | 0.436 | **0.519** | 0.355 |  | 0.525 | 0.661 |  |
| **Total subscale Cronbach’s Alpha** |  |  |  |  |  | **0.727** |  |
|  |  |  |  |  |  |  |  |
| ***Calorie control*** |  |  |  |  |  |  |  |
| 1. Order a small meal at fast food restaurants | 0.429 | 0.225 | **0.623** |  | 0.448 | 0.537 |  |
| 1. Use a little bit of mayonnaise on my plate | -0.105 |  | **0.594** | 0.295 | 0.297 | 0.648 |  |
| 1. Order the salad dressing on the side and use a small amount only | 0.425 |  | **0.574** | 0.226 | 0.474 | 0.518 |  |
| 1. Eat more often white cheeses instead of yellow cheeses | 0.329 |  | **0.484** | 0.234 | 0.441 | 0.542 |  |
| **Total subscale Cronbach’s Alpha** |  |  |  |  |  | **0.632** |  |
|  |  |  |  |  |  |  |  |
| ***Adherence to dietary guidelines*** |  |  |  |  |  |  |  |
| 1. Eat fruits I like during the day with one or more meals |  | 0.182 |  | **0.736** | 0.438 | 0.449 |  |
| 1. Eat vegetables I like during the day with one or more meals |  | 0.124 | 0.204 | **0.722** | 0.449 | 0.426 |  |
| 1. Eat breakfast daily | 0.135 |  | 0.237 | **0.607** | 0.332 | 0.608 |  |
| **Total subscale Cronbach’s Alpha** |  |  |  |  |  | **0.595** |  |

Factor loading less than 0.1 were excluded from the table for simplicity

**Table 5**. Rotated factor loading matrix, Item-to-total score correlation and internal consistency for the practices scale items.

|  | Factor 1 | Factor 2 | Factor 3 | Corrected Item-Total Correlation | Alpha if Item Deleted | Alpha for total scale |
| --- | --- | --- | --- | --- | --- | --- |
| **Practices items** |  |  |  |  |  |  |
| ***Adherence to dietary guidelines*** |  |  |  |  |  | **0.792** |
| 1. Eat 4-5 different types of fruits and vegetables and grains and legumes every week | **0.660** |  |  | 0.490 | 0.728 |  |
| 1. Eat fruits and veg daily with meals and snacks | **0.590** |  |  | 0.461 | 0.731 |  |
| 1. Check the food label for energy fat and sugar content | **0.546** | 0.440 |  | 0.510 | 0.723 |  |
| 1. Choose grilled or baked products | **0.526** | 0.327 |  | 0.450 | 0.732 |  |
| 1. Select fish chicken without skin lean meat legumes like lentils beans | **0.523** |  | -.383 | 0.338 | 0.746 |  |
| 1. Choose whole grain | **0.502** |  |  | 0.378 | 0.741 |  |
| 1. Choose homemade pastries and biscuits instead of packaged pastry or biscuits | **0.483** |  |  | 0.364 | 0.742 |  |
| 1. Share a meal with a friend at fast food restaurant | **0.466** |  |  | 0.351 | 0.744 |  |
| 1. Drink water and fresh natural juices instead of soda and bottled juices | **0.460** | 0.338 | .300 | 0.477 | 0.728 |  |
| 1. Eat breakfast every day | **0.458** |  |  | 0.267 | 0.758 |  |
| 1. Replace whole milk &dairy with low fat milk& dairy | **0.455** |  | .409 | 0.393 | 0.740 |  |
| **Total subscale Cronbach’s Alpha** |  |  |  |  | **0.756** |  |
|  |  |  |  |  |  |  |
| ***Salt and sugar food choices*** |  |  |  |  |  |  |
| 1. Eat from mixed nuts or chips or pickles or crackers or fast food fries |  | **0.733** |  | 0.479 | 0.474 |  |
| 1. When I want to eat chips I take one handful from the chips bag |  | **0.616** |  | 0.333 | 0.541 |  |
| 1. Eat foods like chocolate, cakes, biscuits and sweets |  | **0.560** |  | 0.213 | 0.619 |  |
| 1. Choose low calorie ice cream sweets and deserts |  | **0.489** |  | 0.385 | 0.512 |  |
| 1. Use the salt shaker at the table to season my food |  | **0.483** |  | 0.367 | 0.522 |  |
| **Total subscale Cronbach’s Alpha** |  |  |  |  | **0.589** |  |
|  |  |  |  |  |  |  |
| ***Lifestyle and portion size*** |  |  |  |  |  |  |
| 1. When I want to have a desert I will buy only one scoop of ice cream or request that half my bowl be filled |  |  | **0.756** | 0.348 | 0.277 |  |
| 1. When I want to have a chocolate for a snack I take half a bar |  | 0.375 | **0.657** | 0.324 | 0.325 |  |
| 1. Eat while watching TV |  |  | **0.334** | 0.218 | 0.500 |  |
| **Total subscale Cronbach’s Alpha** |  |  |  |  | **0.475** |  |

Factor loading less than 0.3 were excluded from the table for simplicity

Items 12,14,16,19 were coded inversely before running factor analysis
